# Effects of mechanical abrasion challenge on sound and demineralized dentin surfaces treated with SDF

**DOI:** 10.1038/s41598-020-77035-9

**Published:** 2020-11-16

**Authors:** Mahmoud Sayed, Yuka Tsuda, Khairul Matin, Ahmed Abdou, Kim Martin, Michael F. Burrow, Junji Tagami

**Affiliations:** 1 Department of Cariology and Operative Dentistry, Graduate School of Medical and Dental Sciences, Tokyo Medical and Dental University (TMDU), 1-5-45, Yushima, Bunkyo-ku, Tokyo, 113-8549 Japan; 2 Endowed Department of International Oral Health Science, Tsurumi University, Kanagawa, Japan; 3 Biomaterials Department, Faculty of Oral and Dental Medicine, Modern University for Technology and Information, Mokatam, Cairo, Egypt; 4 Department of Operative Dentistry and Periodontology, University Hospital, LMU Munich, Munich, Germany; 5 Faculty of Dentistry, University of Hong Kong, Hong Kong, SAR China

**Keywords:** Dentistry, Disease prevention, Health care economics, Public health

## Abstract

This study evaluated the effect of mechanical abrasion on the surface integrity, color change (ΔE) and antibacterial properties of demineralized and sound dentin surfaces treated with silver-diammine-fluoride (SDF). The dentin specimens were divided into two groups: sound and demineralized dentin, then divided into three sub-groups, control (no-treatment), SDF, and SDF + potassium-iodide (KI). Each sub-group was further divided into two groups, one exposed to mechanical brushing and the other without brushing. Specimens were analyzed for the ΔE, surface roughness/surface loss and antibacterial properties (CFU, optical density and fluorescent microscope). Repeated Measures ANOVA was used for statistical analysis of color change while one-way ANOVA was used for CFU analysis. SDF and SDI + KI groups showed significant reduction in ΔE with brushing in the sound dentin group unlike the demineralized group. The surface roughness values were higher for both SDF and SDF + KI groups but roughness values significantly decreased after brushing. Both SDF and SDF + KI groups revealed significantly less surface loss than control. The SDF group showed high anti-bacterial effect after brushing, unlike SDF + KI group. So, we concluded that mechanical brushing improved the esthetic outcome. While, SDF and SDF + KI could protect the dentin surface integrity. SDF-treated dentin possesses an antibacterial property even after mechanical brushing.

## Introduction

A high prevalence of dental caries among different age groups and populations remains, despite the existence of multiple global prevention programs. As the philosophy of caries management has changed from a surgical to a medical model, the use of fluoride and other anti-caries agents has become more prominent^1^.

Among the fluoride containing materials, silver diammine fluoride (SDF) is regaining interest among researchers and dental clinicians. SDF possesses a significant antibacterial property especially against *S. mutans* and other cariogenic bacteria in addition to its unique ability to arrest caries and to simultaneously prevent the formation of new carious lesions^2^. These characteristics make SDF different from other caries-preventive agents. The effectiveness of SDF has been shown by clinical trials reporting the ability of SDF to arrest coronal^3^ and root caries lesions^4^. A meta-analysis showed an overall rate for arresting caries at 81% after using SDF^5^.

Additionally, SDF is a non-invasive, simple and inexpensive treatment^6,7^ that can provide care to those with dental fears as well as pediatric populations. SDF has been suggested as a method to arrest or prevent caries in at-risk populations, such as geriatric, the disabled or socially deprived cases^8,9^.

Despite the higher clinical efficacy of SDF, the resulting black stain is a concerning side effect of SDF application and affects patients’ and/or parents’ acceptance of this treatment^10,11^.

The use of a supersaturated solution of potassium iodide (KI) after SDF application, has been promulgated as a solution to overcome the staining problem. However, recently it was noticed that the color improvement with KI application is only temporary and the darkening of tooth surfaces still occurs^12^.

Many concerns were raised about the high fluidity of SDF solution as it may become diluted or washed away easily after application^13^. Thus, the objective of this study was to evaluate the effect of mechanical abrasion on the surface integrity of demineralized and sound dentin surfaces, color change and antibacterial properties after application of SDF. The null hypothesis was that mechanical brushing will not remove SDF discoloration, and there will be no surface tissue loss and no antibacterial effect.

## Materials and methods

This study protocol was approved by the ethics committee of Tokyo Medical and Dental University under identification code “D2013-022-02” (Institutional Research Board approval number: 725). All methods in this study were performed in accordance with the relevant guidelines and regulations.

### Specimen preparation

A total of 270 dentin specimens were used in this study. The specimens (6 × 6 × 2 mm) were obtained from the cervical portion of bovine incisor roots. Teeth were cut horizontally right below the cemento-enamel junction, then the cervical portion of the root was cut vertically to obtain dentin specimens using a low-speed diamond saw (Isomet 1000, Buehler, IL, USA) under copious water coolant. Specimens were embedded in acrylic-resin (Unifast III, GC, Tokyo, Japan) and the surfaces underwent a standardized polishing regime using a series of silicon-carbide papers from 600 to 2000-grit (Fuji Star, Sankyo Rikagaku, Saitama, Japan) under running water, then ultra-sonicated in distilled water (DW) (Milli-Q water; Millipore, USA) for 3 min.

The specimens were randomly assigned into two groups: sound and demineralized dentin groups. The exposed dentin surfaces were finally covered using nail varnish (Revlon, New York, USA) leaving a 5 × 5 mm^2^ window. On each specimen, the nail-varnish covered area served as the sound reference. For the demineralized group, the specimens were demineralized with 0.5 M ethylene diamine tetra-acetic acid adjusted to pH 7.5 (EDTA: decalcifying Soln. B, Wako Pure Chemical Industries, Osaka, Japan) for 13 h resulting in a 180 ± 3.5 µm deep zone of demineralization^14^. Each group was divided into three sub-groups according to surface treatment: (1) control (no-treatment), (2) 38% SDF (Saforide, Bee-Brand Medico Dental, Tokyo, Japan), and (3) SDF + KI (Riva Star, Southern Dental Industries, Victoria, Australia). All specimens were incubated at 37 °C in artificial saliva (AS) “prepared within 24 h before use and consisted of calcium chloride dihydrate 0.7 mmol/l, magnesium chloride 0.2 mmol/l, potassium dihydrogen phosphate 4.0 mmol/l, HEPES [4-(2-hydroxyethyl)-1-piperazineethanesulfonic acid buffer] 20.0 mmol/l and potassium chloride 30.0 mmol/l and buffered to pH 7^15^” for 48 h (until a significant dentin discoloration occurred).

Each sub-group was then divided into a further two groups, one exposed to mechanical brushing and the other kept without brushing. In this regard, 120 specimens were used for the color assessment test, surface roughness and surface loss respectively (n = 10). Sixty specimens were used for the scanning electron microscope/electron diffraction spectroscopy (SEM/EDS) analysis (n = 5). In addition, 90 specimens “subjected to mechanical brushing” were prepared for the antibacterial test.

### Treatment protocols

#### SDF

The application protocol for 38% SDF was performed following the manufacturer’s instructions. Specimens were air-dried then 1–2 drops of SDF was added to a mixing-well and applied on the dentin surfaces using a micro-brush for 1 min. The specimens were left for 2 min then rinsed with distilled water for 30 s.

#### SDF + KI

Specimens were air dried, then 1 drop of SDF was applied with a micro-brush for 1 min. Immediately following the application of SDF, a saturated KI solution was applied until creamy-white precipitates turned into clear, and then washed with copious amounts of distilled-water for 30 s.

### Brushing procedures

A commercially available toothbrush (Prospec toothbrush-Young, GC, Tokyo, Japan) with medium hardness, flat trim and nylon filaments (diameter of 0.2 mm) was used for the brushing procedures.

Brushing was performed using an automatic brushing machine (K236, Tokyo Giken, Tokyo, Japan). The toothbrush was secured parallel to the specimen surface in the brushing machine. Specimens were fully immersed in a dentifrice slurry in reservoir baths located in the tooth brushing machine and were brushed with 60 strokes/cycle (2 cycles/days) under a load of 250 g^16^. The brushing procedure started on the third day after the surface treatment and continued for 7 days. The dentifrice slurry was prepared immediately before use. The slurry was diluted using a 1-part fluoride dentifrice (CLINICA, Lion, Tokyo, Japan, 950 ppm F) and 3-parts distilled water^17^. After brushing, the samples were rinsed with distilled water until all visible remnants of toothpaste were removed. The toothpaste slurry was replaced after each cycle, while the same toothbrush head was used for the same specimen throughout the study. In order to fix the specimen in the automatic brushing machine a resin composite plate was used as a guide. The specimens were kept in artificial saliva after each brushing cycle.

### Color assessment

One hundred and twenty specimens were used in this test (n = 10). Color assessments of the dentin specimens were recorded at different time-interval points: baseline (before surface treatment), immediately after surface treatment, then at 2, 3, 7 and 10 days. The brushing procedure started on the third day after the surface treatment. The color and photographs of the dentin surfaces were recorded using a spectrophotometer (Crystaleye M639001, Olympus, Tokyo, Japan). The spectrophotometer was calibrated before each examination time according to the manufacturer’s instructions. Each color record was acquired using the 3-dimensional color space system CIELAB (L*a*b* system), where L* represents brightness ranging from bright (100) to dark (0), a* describes green (− a*) to red (+ a*), and the b* represents blue (− b*) to yellow (+ b*). The color measurements were replicated three times for each specimen at each time period by single operator “who was blinded to the active ingredient of the experimental groups” and the mean values were recorded^18^. The difference in color (ΔE) for each specimen between baseline and each time-interval point was calculated using this equation;

$$ \Delta {\text{E}}\, = \,[(\Delta {\text{L}})^{{2}} \, + \,(\Delta {\text{a}})^{{2}} \, + \,(\Delta {\text{b}})^{{2}} ]^{{{1}/{2}}} $$

### Surface roughness analysis

All the specimens were imaged and analyzed for surface roughness by the same operator who was blinded to the active ingredient of the experimental groups. Surface scanning was done using 3-D confocal scanning laser microscope (CLSM) (Keyence VK-X150, Osaka, Japan) at 5000× magnification. Areas free of dentifrice contamination were randomly selected within the center of the specimen then imaged and analyzed at different time-interval points: baseline (before surface treatment), immediately, 2 days, 3 days, 7 days and 10 days after surface treatment, while the brushing procedure started on the third day after the surface treatment. The data were analyzed using analyzer program (MultiFileAnalyzer V1.3.1.120, Osaka, Japan).

### Surface loss analysis

After the brushing procedures, the nail varnish was removed gently from the dentin surfaces. Then, the specimens were scanned using a 3-D confocal scanning laser microscope (CLSM) (Keyence VK-X150, Osaka, Japan) at 1000× magnification by the same operator who was blinded to the active ingredient of the experimental groups. A 3-D image displayed as a topographic map, where various colors denoted different heights for the image elements. The data were analyzed using an analysis program (MultiFileAnalyzer V1.3.1.120, Osaka, Japan).

### SEM/EDS analysis observation

Sixty specimens were used for this test (n = 5). The specimens were fixed with 2.5% glutaraldehyde for 2 h at 4 °C “primary fixation”, followed by 0.1% osmium solution for 2 h at 4 °C “secondary fixation”, and lastly dehydrated in an ascending ethanol concentration series (50%, 70%, 80%, 90% and 95%) each concentration for 25 min and twice in the 100% for 25 min each, and sputter-coated with carbon^14^. The surfaces of the prepared specimens were examined under SEM (JSM-IT 100, Joel, Tokyo, Japan) with energy dispersive X-ray spectroscopy (EDS) by the same operator who was blinded to the active ingredient of the experimental groups. The specimens were analyzed with SEM under operating conditions of 20 kV. The surface area and point analysis were performed for detection of phosphorous (P), calcium (Ca), silver (Ag) and Iodine (I) ion levels.

### Anti-bacterial test procedures

Thirty specimens “subjected to mechanical brushing” in six groups were used for this test (n = 5) and was repeated three times (total of 90 specimens). Following the method used by Sayed et al., *Streptococcus mutans* MT8148 (*S. mutans*) was used after freshly preparing a suspension (described below) to incubate the samples. After brushing procedures, each specimen was immersed in 150 μl of bacterial suspension in a separate well of a sterile 96-well flat-bottom culture plate^19^.

#### Bacterial suspension preparation

*Streptococcus mutans* were freshly cultured in brain heart-infusion broth (BHI; BD Biosciences, Franklin Lakes, NJ, USA) for 16 h and washed for three times with sterile phosphate-buffered saline (PBS). Then, a suspension of *S. mutans* in PBS was ready to use.

Then, the optical density of the bacterial suspension was corrected by adding up PBS until an optical density of 490 nm (OD_490_) = 0.5 [approximately 3.6 × 10^8^ colony forming unit (CFU)/ml] was recorded using a spectrophotometer (Model 680 Microplate Reader; Bio-Rad, Hercules, CA, USA). After that, the suspensions were used directly after placing 150 μl aliquots into each well of the 96-well culture plate, and incubated at 37 °C for 2 h. All the specimens were transferred into separate dark-colored microtubes for performing the bacterial viability test. The remaining suspensions of the *S. mutans* were used for colony-formation test and growth curve test (OD_490_) as mentioned by Sayed et al.^19^. All the following tests were performed by the same operator who was blinded to the active ingredient of the experimental groups.

#### Bacterial viability test

For the evaluation of the *S. mutans* viability based on the immediate effects of the SDF application on the dentin surfaces after mechanical abrasion, a LIVE/DEAD BacLight Bacterial Viability Kit (Thermo Fisher Scientific, Waltham, USA) was used. After the 2-h incubation period, each specimen in the dark-colored micro-tube “with bacterial-suspension attached on the surface” was stained using 0.5-μl BacLight stain (a mixture of propidium iodide and SYTO-9). Within this staining system, the viable bacterial cells exhibit a green-color fluorescence, while the non-viable cells exhibit a red-color fluorescence. Selective dye uptake by bacteria depends upon the cell membrane integrity, facilitating the dead bacteria to be easily distinguished from viable bacteria. The excitation wavelengths for the dyes were approximately 480/530 nm for SYTO-9 (green-signals) and 520/580 nm for propidium-iodide (red-signals). Bacterial cells viability was evaluated using a fluorescence microscope (FM, CKX41; Olympus, Tokyo, Japan) as mentioned by Sayed et al.^19^.

#### Colony forming unit (CFU/ml) count

To observe the effect of the test materials including SDF on the regeneration potential of *S. mutans,* CFU was counted and calculated. From the remaining bacterial suspensions 10 μl each was collected and serially diluted with sterile PBS. Then, they were plated in petri-dishes containing Mitis Salivarius agar medium. After 48-h incubation at 37 °C under anaerobic conditions, the number of CFU/ml was counted using an optical microscope as mentioned by Sayed et al.^19^.

#### Growth curve of the bacteria (OD_490_)

After removing the specimens from the bacterial suspension, 150 μl of an autoclaved solution of BHI was added to each well that contains the bacterial suspensions and incubated at 37 °C. Then incubated at 37 °C optical density was immediately measured as baseline and then continued to be measured hourly for 8 h using the same spectrophotometer at 490 nm as mentioned by Sayed et al.^19^.

### Statistical analysis

Data were analyzed for normality using the Kolmogorov–Smirnov and Shapiro–Wilk tests. ΔE and surface roughness showed a parmatric distribution, so repeated Measures ANOVA test was used to compare the effect of substrate (Sound and demineralized dentin), Brushing (mechanical brusching or without brushing), and materials (no treatment [control], 38% SDF, and SDF + KI) at different time intervals (immediately and after 7, 8, 10, and 14 days) on the ΔE and surface roughness. The test was followed by pairwise comparison with Bonferroni correction. The correlation between surface loss and ΔE was done using pearson correlation coefficient. For CFU and surface loss, data showed a prametric distribution so two-way ANOVA was used to compare tested materials and substrate followed by multiple comparison with Tukey TSD test. The significant level was set at 0.05 (α = 0.05). Statistical analysis was performed using statistical package for social scineces (SPSS Inc., IBM, Armnok, NY, USA) Statistics Version 23 for Windows. The power analysis for this study was performed using “PASS 15 Power Analysis and Sample Size Software (2017), NCSS, LLC. Kaysville, USA” (Supplementary Data S1).

## Results

### Spectrophotometric measurement of color change

Repeated measure ANOVA showed that the substrate, brushing, materials and time intervals and all their possible interactions had a significant effect on the ΔE (*p* < 0.001). The multiple comprsions analysis showed that in the demineralized dentin group, SDF groups recorded the greatest color change values compared with SDF + KI and control groups with and without brushing at all time intervals. The color parameter that changed most was ΔL (representing greater darkening of the specimen) in the SDF group, while the Δa was the greatest changed color parameter in the SDF + KI group (representing more yellowish-discoloration of the specimen). However, the pattern of ΔE was different. ΔE in both SDF groups (with and without brushing) significantly increased up to 2 days, then gradually continued increasing in the SDF without brushing group while significantly decreasing in the SDF + brushing group once brushing procedures started after 2 days of application. In case of SDF + KI, showed stability in color change with no significant changes over time in the non-brushing group. On the other hand, SDF + KI color change decreased significantly after brushing (Table 1) (Supplementary Figure S1).

**Table 1:** Mean and standard deviation (SD) of the ΔE values for sound and demineralized dentin surfaces treated with different materials at different time intervals.

	Demineralized			Sound		
	Control	SDF	SDF + KI	Control	SDF	SDF + KI
**B−**						
Immediate	0.17 ± 0.02^fD^	25.74 ± 0.5^eA^	14.69 ± 0.59^bB^	0.25 ± 0.1^eD^	3.45 ± 0.2^fC^	14.92 ± 0.33^bB^
2 days	10.9 ± 0.25^cE^	44.58 ± 0.3^cA^	21.03 ± 0.53^aC^	2.53 ± 0.21^bcF^	32.09 ± 0.85^cB^	13.94 ± 0.16^bcD^
3 days	12.14 ± 0.15^bE^	44.6 ± 0.77^cA^	20.66 ± 0.47^aC^	3.28 ± 0.3^aF^	32.87 ± 0.78^bcB^	14.21 ± 0.36^bD^
7 days	12.91 ± 0.42^bE^	46.39 ± 0.48^bA^	20.4 ± 0.4^aC^	2.9 ± 0.48^abcF^	33.75 ± 0.79^bcB^	14.92 ± 0.33^bD^
10 days	14.91 ± 0.59^aE^	48.43 ± 1.35^aA^	21.49 ± 0.88^aC^	3.02 ± 0.56^abF^	37.34 ± 0.8^aB^	17.64 ± 0.62^aD^
**B+**						
Immediate	0.17 ± 0.05^fD^	25.63 ± 0.33^eA^	14.31 ± 0.57^bB^	0.19 ± 0.04^eD^	3.45 ± 0.22^fC^	14.63 ± 0.59^bB^
2 days	10.83 ± 0.38^cE^	45.33 ± 0.51^cbA^	20.79 ± 0.8^aC^	2.9 ± 0.21^abcF^	31.67 ± 0.42^cB^	13.95 ± 0.48^bcD^
3 days	10.83 ± 0.38^cE^	40.27 ± 1.09^dA^	12.83 ± 0.47^cC^	1.75 ± 0.3^dF^	18.35 ± 0.39^eB^	9.08 ± 0.54^eD^
7 days	7.71 ± 0.53^eD^	40.06 ± 1.29^dA^	11.58 ± 0.75^cC^	2.45 ± 0.37^bcE^	18.58 ± 0.8^eB^	11.23 ± 0.77^dC^
10 days	9.06 ± 0.73^dD^	41.24 ± 0.87^dA^	11.78 ± 0.57^cC^	2.28 ± 0.3^cdE^	21.01 ± 1.33^ dB^	13.03 ± 0.5^cC^

Different lowercase letter within each row indicates significant difference, different uppercase letter within each column indicates significance difference (p < 0.05). “B−” means “without brushing” and “B+” means “with brushing”.

The sound dentin groups showed the same pattern as the demineralized groups, however at lower values of ΔE than the SDF group showing improvement in the esthetic outcome with brushing. (Table 1) (Supplementary Figure S2).

### Surface roughness analysis

Repeated measure ANOVA showed that the substrate, brushing and materials had a significant effect on the mean surface roughness (*p* < 0.05). Conversely, the interaction between materials + substrate, substrate + brushing and materials + substrate and brushing were not significant (*p* = 0.059, 0.215 and 0.325, respectively). The multiple comprsions analysis showed that in the demineralized group, there was no statistical significance between groups immediately after material application. However, after 2 days the surface roughness increased dramatically in the SDF and SDF + KI groups compared with control group. In the non-brushing group, SDF continued to show an increase in roughness values while SDF + KI showed more stable values. In the brushing group, SDF group showed a significant decrease in the roughness values while SDF + KI group roughness values remained higher than other groups.

The sound dentin group showed the same pattern as the demineralized groups regarding the non-brushing group. However, in the brushing group, both SDF and SDF + KI groups showed a significant decrease in the roughness values after brushing procedures with no significant difference between them after 14 d. (Table 2).

**Table 2:** Mean and standard deviation (SD) of the roughness values for sound and demineralized dentin surfaces treated with different materials at different time intervals.

	Demineralized			Sound		
	Control	SDF	SDF + KI	Control	SDF	SDF + KI
**B−**						
Immediate	0.188 ± 0.031^cA^	0.227 ± 0.048^efA^	0.202 ± 0.043^bA^	0.086 ± 0.011^aA^	0.086 ± 0.013^cA^	0.086 ± 0.021^aA^
7 days	0.345 ± 0.074^abcB^	0.688 ± 0.174^abA^	0.673 ± 0.172^aA^	0.112 ± 0.012^aC^	0.251 ± 0.073^abcBC^	0.194 ± 0.062^aBC^
8 days	0.353 ± 0.08^abcB^	0.707 ± 0.175^abA^	0.673 ± 0.166^aA^	0.115 ± 0.019^aC^	0.257 ± 0.065^abcBC^	0.205 ± 0.046^aBC^
10 days	0.378 ± 0.075^abcB^	0.745 ± 0.165^abA^	0.676 ± 0.164^aA^	0.126 ± 0.031^aC^	0.288 ± 0.049^abBC^	0.22 ± 0.044^aBC^
14 days	0.492 ± 0.069^aB^	0.776 ± 0.156^aA^	0.687 ± 0.156^aA^	0.136 ± 0.024^aD^	0.324 ± 0.057^aBC^	0.232 ± 0.04^ACD^
**B+**						
Immediate	0.242 ± 0.056^bcA^	0.205 ± 0.067^fA^	0.196 ± 0.053^bA^	0.083 ± 0.007^aA^	0.089 ± 0.026^cA^	0.1 ± 0.006^aA^
7 days	0.404 ± 0.104^abBC^	0.567 ± 0.132^bcAB^	0.664 ± 0.177^aA^	0.124 ± 0.011^aD^	0.246 ± 0.051^abcCD^	0.258 ± 0.063^aCD^
8 days	0.413 ± 0.094^abB^	0.442 ± 0.107^cdeAB^	0.614 ± 0.187^aA^	0.117 ± 0.017^aC^	0.155 ± 0.015^abcCD^	0.189 ± 0.037^aCD^
10 days	0.438 ± 0.09^aAB^	0.407 ± 0.126^cdeB^	0.589 ± 0.207^aA^	0.151 ± 0.033^aC^	0.137 ± 0.017^abcCD^	0.156 ± 0.033^aCD^
14 days	0.458 ± 0.085^aAB^	0.356 ± 0.077^defBC^	0.558 ± 0.204^aA^	0.215 ± 0.069^aCD^	0.128 ± 0.022^bcD^	0.136 ± 0.03^aD^

Different lowercase letter within each row indicates significant difference, different lowercase letter within each column indicates significance difference (*p* < 0.05).

### Surface loss analysis

**Figure 1:**
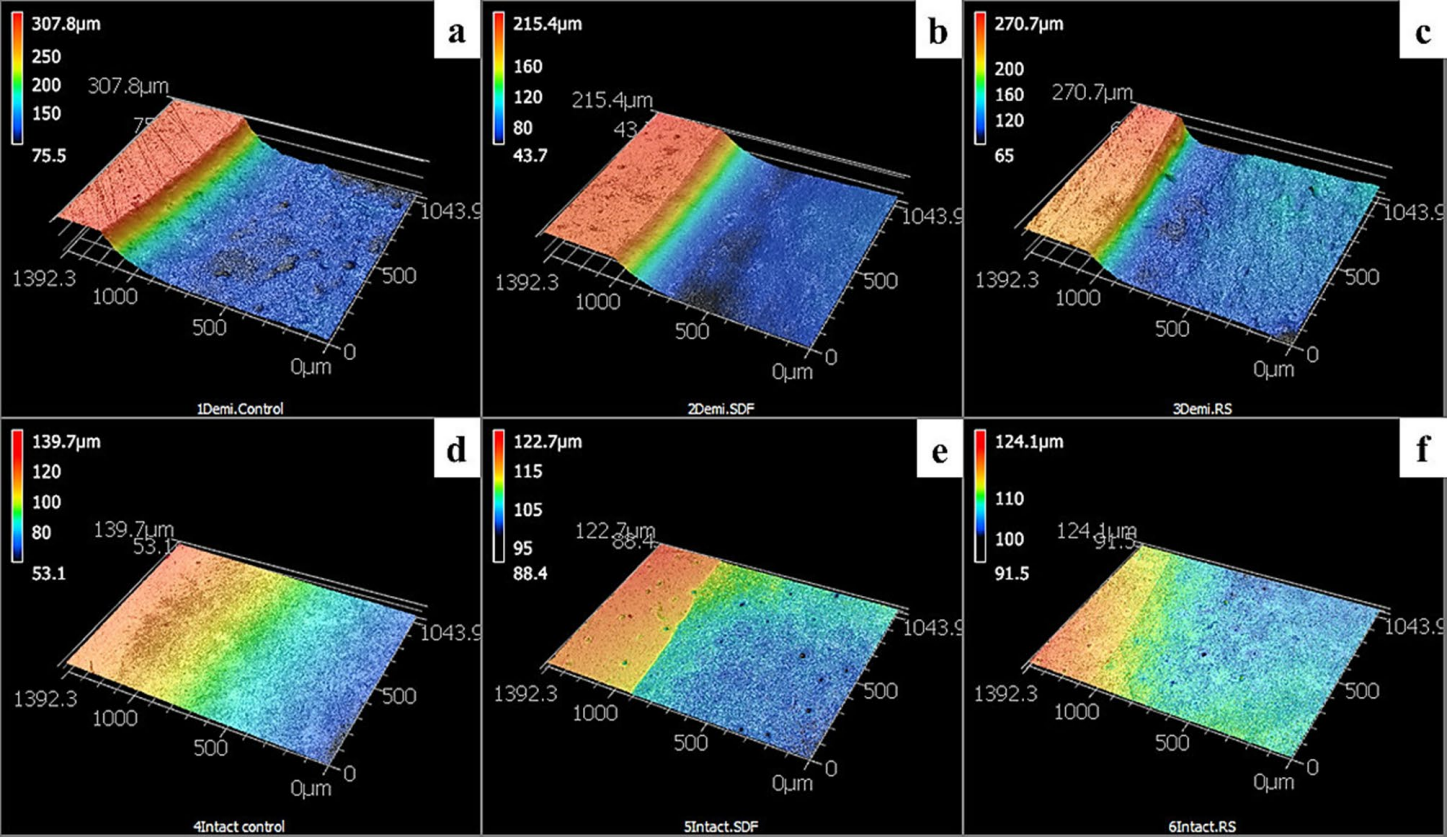
Representative 3D-CLSM (confocal laser scanning microscope) images showing the surface loss of sound and demineralized dentin specimens treated with different materials, after brushing procedures, Demineralized dentin: (**a**) control, (**b**) SDF, (**c**) SDF + KI, sound dentin: (**d**) control, (**e**) SDF, (**f**) SDF + KI.

Two-way ANOVA showed that both the substare and materials in addition to the interaction between them had a significant effect on the mean CFU/ml (p < 0.001). After the brushing procedures, SDF + KI group recorded the lowest surface loss values (26.7 µm) followed by SDF group (22 µm) and lastly control group (39 µm) in sound dentin with a significant difference determined between the groups. The same pattern was noticed in the demineralized dentin groups, with SDF + KI group recording the lowest surface loss values (88.4 µm) followed by SDF group (129.5 µm) and lastly the control group (213.9 µm) with significant difference noted among the groups (Fig. 1; Table 3).

**Table 3:** Mean and standard deviation (SD) of the Surface loss after mechanical brushing.

	Con		SDF		SDF + KI	
	Mean	SD	Mean	SD	Mean	SD
Demineralized	213.9^a^	6.7	129.5^b^	35.9	88.4^c^	26.4
Sound	39.0^d^	11.1	22.0^d^	9.8	26.7^d^	12.5

Different letter indicates a significant difference between groups (p < 0.05).

The increase in the surface loss was associated with decrease in the ΔE. The correlation between surface loss and ΔE was significant for the sound dentin substrate (r = − 0.539, p = 0.038), but insignificant for the demineralized dentin substrate (r = − 0.145, p = 0.583).

### SEM/EDS analysis

**Figure 2:**
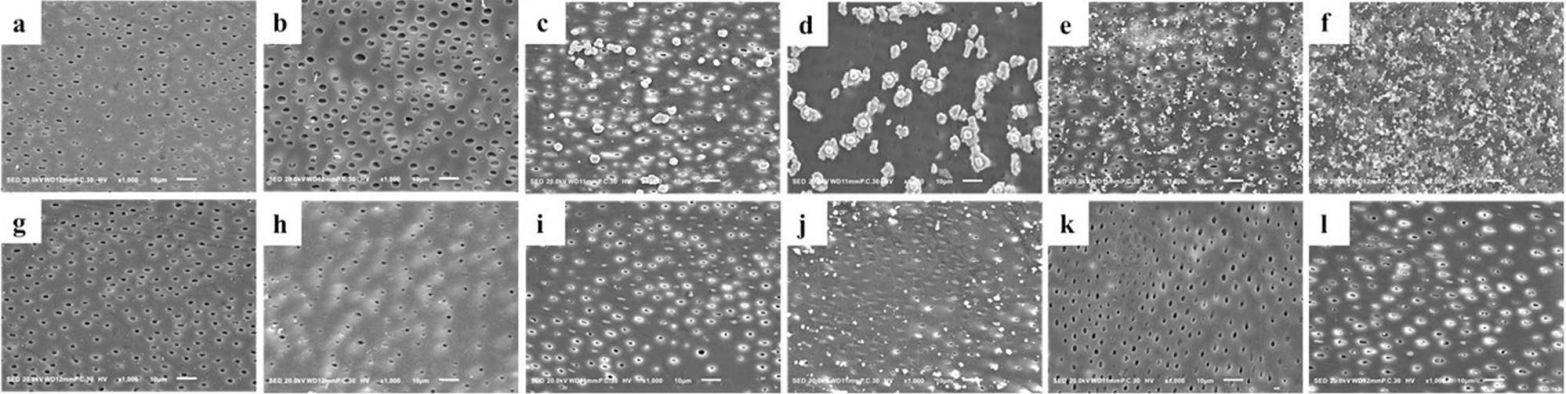
SEM (scanning electron microscope) images showing the surfaces of sound and demineralized dentin specimens treated with different materials, before and after brushing procedures. Before brushing: (**a**) control sound dentin, (**b**) control demineralized dentin, (**c**) SDF-treated sound dentin, (**d**) SDF-treated demineralized dentin, (**e**) SDF + KI-treated sound dentin, (**f**) SDF + KI-treated demineralized dentin. After brushing: (**g**) control sound dentin, (**h**) control demineralized dentin, (**i**) SDF-treated sound dentin, (**j**) SDF-treated demineralized dentin, (**k**) SDF + KI-treated sound dentin, (**l**) SDF + KI-treated demineralized dentin.

The surface morphology observation revealed multiple surface depositions in all tested groups. However, the surface depositions were more marked in the demineralized dentin groups. After brushing, almost all the deposits were removed from the surfaces except for the SDF demineralized group which still had some deposits remaining on the dentin surfaces (Fig. 2).

**Figure 3:**
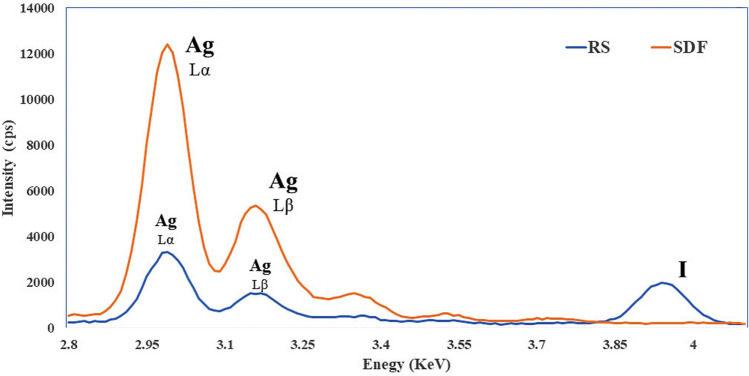
EDS (electron diffraction spectroscopy) analysis for the specimen’s surface depositions in the SDF-treated dentin and SDF + KI-treated dentin.

EDS analysis showed high peaks for Ag and I for the surface deposits of SDF + KI specimens suggesting formation of AgI compounds. While for the surface deposits of SDF specimens, the EDS analysis recorded a high peak for Ag indicating formation of silver compounds (Fig. 3).

### Anti-bacterial test

**Figure 4:**
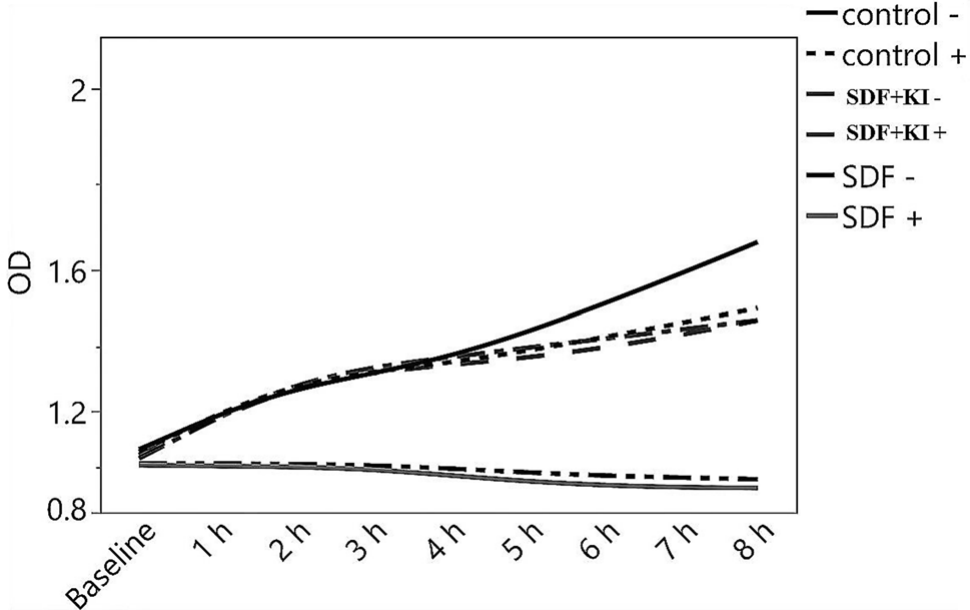
Line chart showing the optical density (OD) values of different tested groups at different time intervals. “+” means sound dentin while “−” means Demineralized dentin.

The results of CFU/mL measurements (Table 4) of *S. mutans.* Two-way ANOVA showed that both the substare and materials in addition to the interaction between them had a significant effect on the mean CFU/ml (p < 0.001). After brushing procedures, a significant bactericidal effect of SDF in both sound and demineralized dentin groups through recording the lowest values in CFU/mL measurement values compared to SDF + KI and control groups (p < 0.001). On the other hand, there was no significant difference between SDF + KI and control groups indicating the low antibacterial properties of SDF + KI after brushing. Regarding OD results (Fig. 4) (Supplementary Table S1), the SDF group showed a plateau of bacterial growth inhibitory phase over time in both sound and dentin groups scoring the lowest OD_490_ values. However, the SDF + KI and the control groups showed an increase in the OD_490_ values over time indicating bacterial growth and a low antibacterial effect of SDF + KI after brushing. Generally, the OD results confirmed the CFU/mL measurements regarding the antibacterial capabilities of the tested materials.

**Table 4:** Mean and standard deviation (SD) of the CFU/ml values obtained from different tested groups.

	Control		SDF		SDF + KI	
	Mean	SD	Mean	SD	Mean	SD
Demineralized	8.85^a^	0.02	6.07^c^	0.33	8.69^a^	0.02
Sound	8.89^a^	0.02	7.40^b^	0.02	8.83^a^	0.01

Different letter indicates a significant difference between groups (p < 0.05).

**Figure 5:**
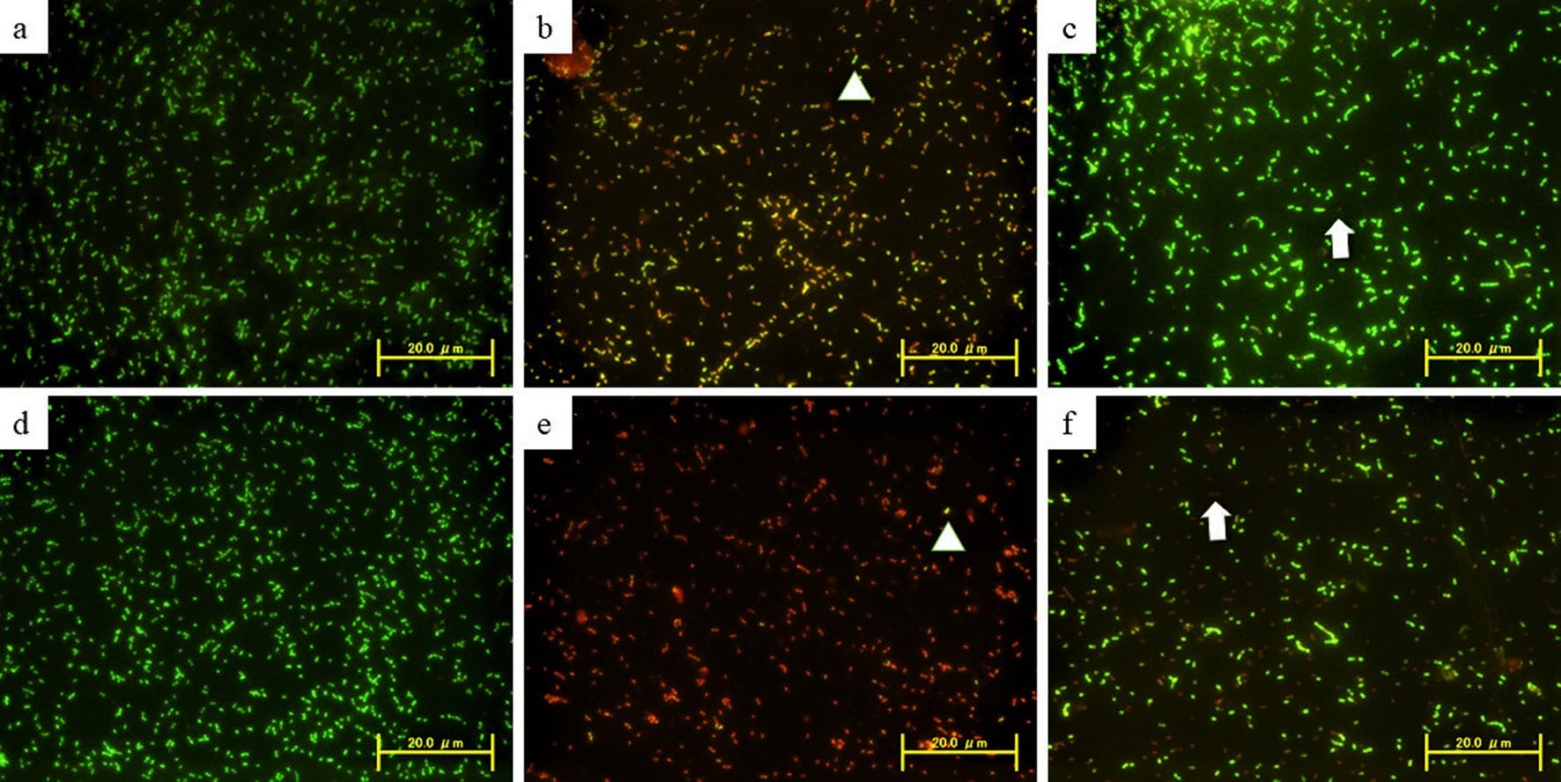
Fluorescence microscopic images showing the viable and dead bacteria in different tested groups. Sound dentin: (**a**) control, (**b**) SDF, (**c**) SDF + KI. Demineralized dentin: (**d**. control, (**e**) SDF, (**f**) SDF + KI. “The viable cells exhibit green fluorescence color, while dead cells exhibit red fluorescence color”.

Fluorescence microscopy observation displayed a strong anti-bacterial effect of SDF represented in multiple cell death of *S. mutans* with most bacteria showing as red in color indicating “dead bacteria”. While SDF + KI and the control groups showed a weak anti-bacterial effect with only a few red bacteria and multiple green active *S. mutans* bacteria detected (Fig. 5).

## Discussion

The present study evaluated the potential mechanical effects of tooth brushing on stain reduction (colour improvement), surface protection and antibacterial properties of sound and demineralized dentin surfaces after the application of silver-containing materials in a laboratory-based study. The results indicated that tooth brushing improved the dentin discoloration more in the case of sound dentin than demineralized dentin after application of SDF. There was a significant difference in roughness change with application of SDF and SDF + KI before and after brushing. In addition, there was evidence of some surface protection created by the application of SDF or SDF + KI compared with the control. Both sound and demineralized dentin proved to have antibacterial properties after mechanical brushing in the SDF-treated dentin group.

In the current study, the dentin was demineralized for 13 h in EDTA solution to simulate the severely-demineralized dentin condition^14^. The load (250 g), brushing time and frequency used in the tooth brushing test were considered to closely replicate the daily situation^16,17^ as oral healthcare measures (e.g. tooth-brushing) are recommended at least twice daily. A spectrophotometer was used in this study for accurate color measurement and as it can use a low light intensity in order to measure the full-visible spectrum through the use of the 3D LAB color space system^6^.

Reactions of SDF with tooth structure should be well-clarified to interpret the results of our study. Upon application of SDF on dentin, it reacts with both the mineral and organic phases of dentin. Regarding the silver reactions with the mineral phase, SDF application results in formation of silver phosphate^20^. However, silver phosphate is an unstable compound and reduces gradually into metallic silver by reducing agents such as light exposure^14^ or replaced by silver chloride after immersion in artificial saliva^21^ and the formation of metallic silver^6,22^. All the previously mentioned silver compounds contribute to hardening and protection of the dentin surface as well as the formation of the dark stains. Concerning fluoride reactions, it can react with the tooth hydroxyapatite in many ways. First, fluoride can be incorporated into the apatite crystals “firmly-attached fluoride” through ion-exchange of fluoride ions for hydroxyl ions^23^ resulting in formation of fluor-hydroxyapatite crystals containing different fluoride percentages^24^. Second, fluoride ions released from SDF react with hydroxyapatite to form a calcium fluoride-like material^25^. However, the formed calcium fluoride (CaF_2_) is adsorbed onto the tooth surface rather than incorporated into it. Therefore, calcium fluoride is considered as a loosely-attached type of fluoride that is easily washed away^20,25^. Calcium fluoride is important because it acts as a temporary reservoir of fluoride ions which release F ions at low pH, promoting remineralization by facilitating formation of fluoroapatite^26,27^. Fluoroapatite is more chemically stable than hydroxyapatite in acidic conditions, making the tooth structure more resistant to dissolution^28^.

Regarding the reactions with dentin organic-phase, part of the silver released from the SDF are attached to the dentin collagen, as the silver ions are considered as a good electron-acceptors that are characterized by a its lower affinity for oxidation and large atomic radius^27^. Moreover, silver aquires a high polarizing-power due to the high ratio of the ionic charge compared with the radius of the ion that in turn aids the formation of strong bonds with sulfur and nitrogen groups found within the histidine and cysteine proteins^29^. This reaction results in formation of a protective layer of silver–protein complex on the surface of a demineralized lesion. Thus, SDF application on exposed-collagen resulted in a relatively quick discoloration, suggesting immediate reduction of the silver ions into metallic silver^6,25^. This can explain the darker-discoloration of the demineralized dentin specimens when compared to the sound ones.

SDF can penetrate deeply into dentin with the highest concentration “represented by darkest zone” being located on the dentin surface^6^. Mechanical brushing has a wearing effect on dentin surfaces, which may result in partial removal of the formed silver compounds from the dentin surface which in turn decreased the dentin discoloration associated with SDF application. However, it was not as effective in the case of demineralized dentin as silver is chemically attached to the exposed collagen. The correlation between surface loss and ΔE showed that the removal of dentin surface layer lowered the discoloration in both sound and demineralized dentin groups, however the decrease in discoloration was significant in the sound dentin group.

Based on the present findings, it can be hypothesized that fluoride and silver components of SDF protected the dentin surface against surface loss^30^ through formation of fluoroapatite, CaF_2_ and insoluble silver products like silver phosphate, silver chloride or metallic silver^31,32^. On the other hand, formation of the insoluble silver compounds on dentin surface may increase the surface roughness of dentin.

Clinically, SDF is recommended as a cost-saving treatment for arresting carious lesions and prevention of root caries in high-risk patients^7^. Application of a saturated KI solution immediately after SDF was suggested as a way to resolve the discoloration problem produced by SDF application. In that case, iodide ions react with the excess free silver ions to form a yellowish precipitate of insoluble silver iodide crystals on the surface^18,33^. Although the formation of the insoluble silver iodide crystals contributed to the high surface roughness of the dentin surface even after brushing, it also resulted in protection of the dentin surface against the wearing effect from brushing resulting in lower surface loss.

Addition of KI improved the color and protected the surface against the wearing effect of brushing. However, it was reported that it did not maintain a long-term effect on improving the aesthetic problem^12^.

The antibacterial effect of SDF has been proven in previous studies^20,34–36^. This potent antibacterial effect is related to the high concentration of silver and fluoride ions in SDF. Silver compounds are ionized in the presence of water or body fluids and release silver ions^37^. Silver ions have several mechanisms to create an antimicrobial effect. That is why it is difficult to develop bacterial resistance against silver. First, silver can bind to bacterial surfaces causing disruption of membrane transport functions and inhibit the movement of the organism or cause the membrane to leak or rupture^38^. Second, silver can react with bacterial DNA, causing mutation of DNA and inhibition of cell division^39^. Third, silver ions are highly reactive and bind to the thiol group (SH) found in the enzymes causing its deactivation and resulting in bacterial cell death^40^. Fourth, silver ions bind to the amino acids forming a protein–metallic complex. Then, when this protein–metallic complex breaks down, the silver ions are generated inside the bacterial cell. The accumulation of silver ions in the cell can inactivate bacterial DNA and RNA, in addition to the damage and rupture of the cell membrane, resulting in cell death^34^. Fluoride also has an antibacterial effect through the direct inhibition of cellular enzymes or by enhancing the proton permeability of cell membranes in the form of hydrogen fluoride^41^. The presence of SDF antibacterial effect even after mechanical brushing is related to deep penetration of silver and fluoride ions into both sound and demineralized dentin^6^ providing an antibacterial effect even if most of the silver and fluoride ions are removed from the dentin surface.

The diminished antibacterial effect of SDF + KI might be related mainly to the reaction of iodide ions with most of the free silver ions to form silver iodide compounds. The silver ions are responsible for the antibacterial effect^42^. Another factor may be related to the reduced antibacterial effect is that the commercial product for SDF + KI “Riva star” contains 30–35% silver fluoride and more than 60% ammonia solution compared with the 38% SDF used in this study, which may affect the outcome^36^.

Based on the findings of this research, the null hypothesis is partially rejected as mechanical brushing improved the color in the case of sound dentin but was not effective in the demineralized dentin. However, the SDF-treated dentin showed an antibacterial effect even after mechanical brushing. More studies should be conducted in the future including the mechanical factors of the SDF-treated dentin and biofilm formation after brushing.

So, it was concluded that the mechanical brushing improved the dentin discoloration more in sound than demineralized SDF-treated dentin, however the dentin surfaces were still discolored. SDF and SDF + KI could protect the dentin surface against mechanical brushing. Both sound and demineralized SDF-treated dentin possessed antibacterial property even after mechanical brushing.

## Supplementary information

Supplementary Information.

Supplementary Figure S1.

Supplementary Figure S2.

Supplementary Table S1.
