# Supplementary material for: Effects of mechanical abrasion challenge on sound and demineralized dentin surfaces treated with SDF
Source: Sci Rep. 2020 Nov 16;10:19884. doi: 10.1038/s41598-020-77035-9 (PMC7669835; doi:10.1038/s41598-020-77035-9)
Supplement: Supplementary file 1 — Supplementary Information. [file 41598_2020_77035_MOESM1_ESM.docx]

**Title:** Effects of Mechanical Abrasion Challenge on Sound and Demineralized Dentin Surfaces treated with SDF.

**Author list:**

Mahmoud Sayed ^a^*, Yuka Tsuda^a^, Khairul Matin^a,b^, Ahmed Abdou^a,c^, Kim Martin^d^, Michael F. Burrow^e^, Junji Tagami^a^

^a^ Department of Cariology and Operative Dentistry, Graduate School of Medical and Dental Sciences, Tokyo Medical and Dental University (TMDU), Tokyo, Japan.

^b^ Endowed Department of International Oral Health Science, Tsurumi University, Kanagawa, Japan

^c^ Biomaterials Department, Faculty of Oral and Dental Medicine, Modern University for Technology and Information, Mokatam, Cairo, Egypt.

^d^ Department of Operative Dentistry and Periodontology, University Hospital, LMU Munich, Germany.

^e^ Faculty of Dentistry, University of Hong Kong, Hong Kong SAR, China.

***Power Analysis***

The power analysis done using “PASS 15 Power Analysis and Sample Size Software (2017). NCSS, LLC. Kaysville, Utah, USA”

***Color change (Delta E)***

Three between variables and 1 within variable has 12 groups with 10 subjects each for a total of 120 subjects. For the within factor, each sample is measured 5 times. This design 100% power for the three between factors if Geisser-Greenhouse Corrected F Test is used with a 5% significance level with the actual standard deviation (SD) were 4.353 (effect size (f)=7.3) for factor B1, 1.00 (f=1.7) for factor B2 and 1.16 (f=1.9) for factor B3. The interaction between B1*B2 and B1*B3 achieved a 100% power with the actual SD were 4.76 (f=7.9) for factor B1*B2 and 4.119 (f=6.9) for factor B1*B3, On the other hand, B2*B3 interaction achieves 17% power with the actual SD were 0.056 (f=0.1). The power of factor W1 and all its interactions with the between variables achieved 100% with the actual SD were 3.986 (f=10.0) for factor W1, 1.991 (f=5.0) for factor W1*B1, 0.853 (f=2.1) for factor W1*B2, and 1.386 (f=3.5) for factor W1*B3.

***Surface Roughness***

The same samples were used for evaluation of the surface roughness after color assessment. So the same repeated measures design were used with 3 between factors and 1 within factor. That design archives 20%, 7% ,and 15% for B1 (SD=0.072, f=0.1), B2 (SD=0.022, f=0.0), and B3 (SD=0.051, f=0.1). The interaction between these variables showed a power of 23% for B1*B2 (SD=0.079, f=0.1), 8% for B1*B3 (SD=0.033, f=0.1), and 7% for B2*B3 (SD=0.024, f=0.0). The within factor (W1) showed a 44% power (SD=0.087, f=0.2). the interactions with the between variables achieve 7% for factor W1*B1 (SD=0.031, f=0.1), 7% for factor W1*B2 (SD=0.023, f=0.1), and 6% for factor W1*B3 (SD=0.017, f=0.0).

***Surface loss***

The two factors of 3 and 2 levels has 6 cells (interactions) with 60 samples (n=10 in each group) achieved a 100% power for each variable and their interaction. Factor A (3 levels) achieved that power at 5% significance level and 57.35 was the actual SD among the groups (f=1.639). Factor B (2 levels) achieved that power at 5% significance level and 29.153 was the actual SD among the groups (f=0.833). Their interaction achieved the 100% power as the actual SD was 23.247 (an effect size of 0.664)

***CFU/ml***

The same 2 factors as in surface loss with 30 sample (n=5 in each group) achieved a power of 96% for factor A (SD=0.233, f=0.778), 100% for factor B (SD=0.980, f=3.267), and 100% power for their interaction B1*B2 (SD=0.295, f=0.984).

***OD test***

OD test was a dependent on the sample size of CFU/ml data
