# Supplementary material for: Effects of mechanical abrasion challenge on sound and demineralized dentin surfaces treated with SDF
Source: Sci Rep. 2020 Nov 16;10:19884. doi: 10.1038/s41598-020-77035-9 (PMC7669835; doi:10.1038/s41598-020-77035-9)

## Slide 1
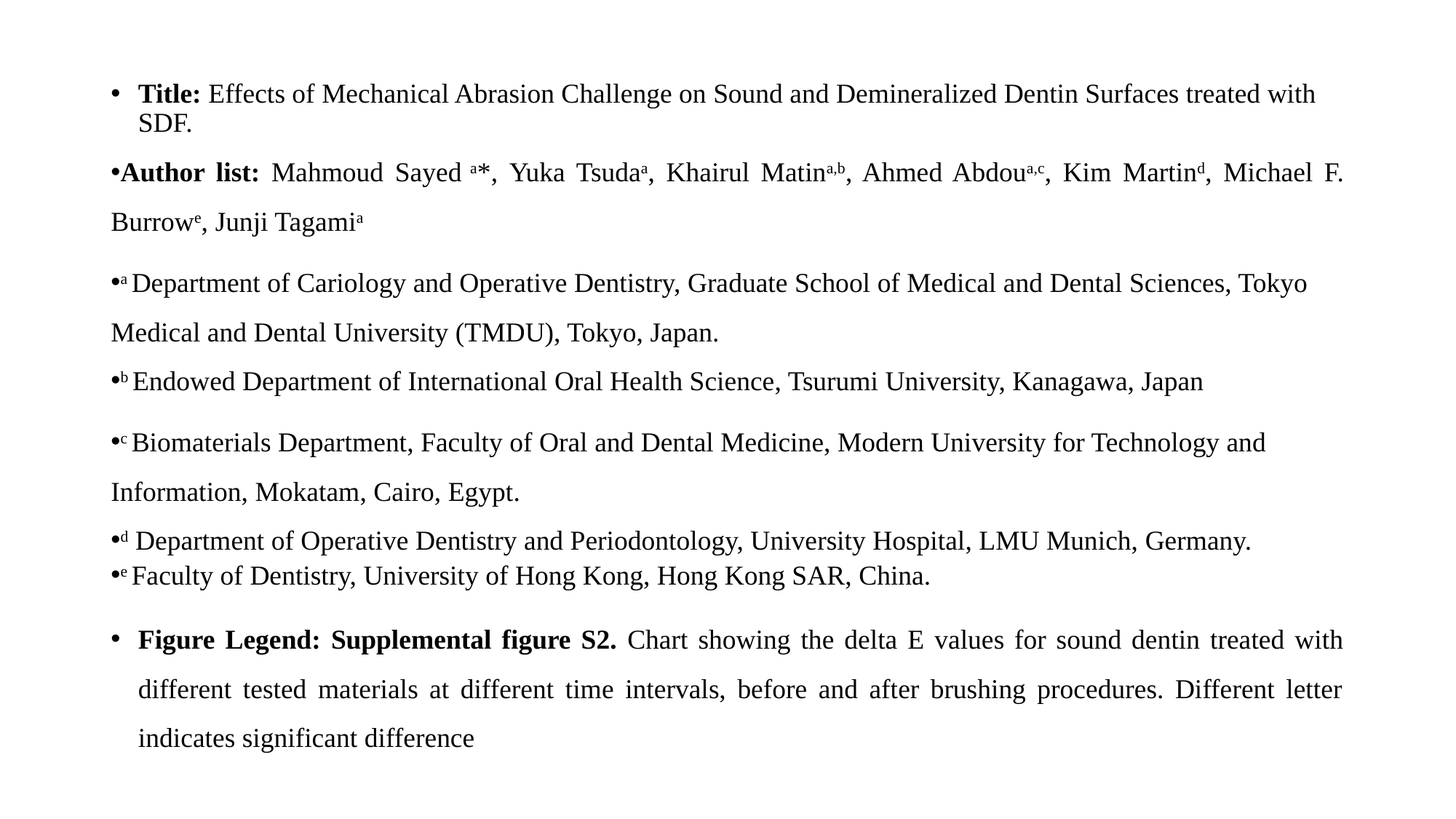

Title: Effects of Mechanical Abrasion Challenge on Sound and Demineralized Dentin Surfaces treated with SDF.
Author list: Mahmoud Sayed a*, Yuka Tsudaa, Khairul Matina,b, Ahmed Abdoua,c, Kim Martind, Michael F. Burrowe, Junji Tagamia
a Department of Cariology and Operative Dentistry, Graduate School of Medical and Dental Sciences, Tokyo Medical and Dental University (TMDU), Tokyo, Japan.
b Endowed Department of International Oral Health Science, Tsurumi University, Kanagawa, Japan
c Biomaterials Department, Faculty of Oral and Dental Medicine, Modern University for Technology and Information, Mokatam, Cairo, Egypt.
d Department of Operative Dentistry and Periodontology, University Hospital, LMU Munich, Germany.
e Faculty of Dentistry, University of Hong Kong, Hong Kong SAR, China.
Figure Legend: Supplemental figure S2. Chart showing the delta E values for sound dentin treated with different tested materials at different time intervals, before and after brushing procedures. Different letter indicates significant difference

## Slide 2
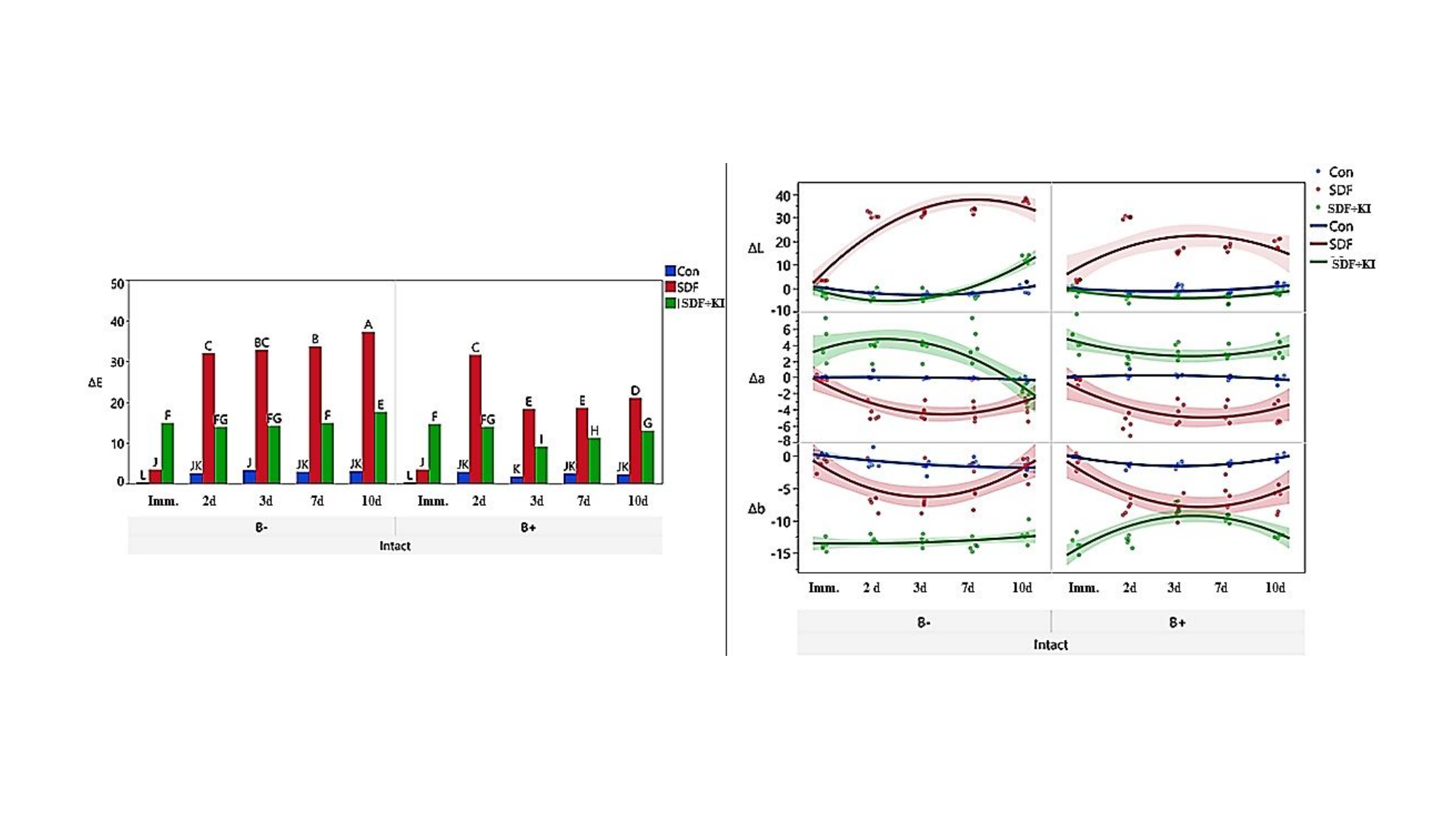

Supplement: Supplementary file 3 — Supplementary Figure S2. [file 41598_2020_77035_MOESM3_ESM.pptx]
