# Supplementary material for: Effects of mechanical abrasion challenge on sound and demineralized dentin surfaces treated with SDF
Source: Sci Rep. 2020 Nov 16;10:19884. doi: 10.1038/s41598-020-77035-9 (PMC7669835; doi:10.1038/s41598-020-77035-9)
Supplement: Supplementary file 4 — Supplementary Table S1. [file 41598_2020_77035_MOESM4_ESM.docx]

**Title:** Effects of Mechanical Abrasion Challenge on Sound and Demineralized Dentin Surfaces treated with SDF.

**Author list:**

Mahmoud Sayed ^a^*, Yuka Tsuda^a^, Khairul Matin^a,b^, Ahmed Abdou^a,c^, Kim Martin^d^, Michael F. Burrow^e^, Junji Tagami^a^

^a^ Department of Cariology and Operative Dentistry, Graduate School of Medical and Dental Sciences, Tokyo Medical and Dental University (TMDU), Tokyo, Japan.

^b^ Endowed Department of International Oral Health Science, Tsurumi University, Kanagawa, Japan

^c^ Biomaterials Department, Faculty of Oral and Dental Medicine, Modern University for Technology and Information, Mokatam, Cairo, Egypt.

^d^ Department of Operative Dentistry and Periodontology, University Hospital, LMU Munich, Germany.

^e^ Faculty of Dentistry, University of Hong Kong, Hong Kong SAR, China.

**Table S1.** Mean and standard deviation (SD) of the OD values for sound and demineralized dentin surfaces treated with different materials at different time intervals

|  | Demineralized | | | Sound | | |
| --- | --- | --- | --- | --- | --- | --- |
|  | Control | SDF | SDF+KI | Control | SDF | SDF+KI |
| Baseline | 1.2±0.04^eA^ | 1.18±0.05^abA^ | 1.21±0.02^dA^ | 1.15±0.02f^A^ | 1.15±0.03^bA^ | 1.16±0.04^eA^ |
| 1 h | 1.37±0.04^cdeAB^ | 1.23±0.04^abC^ | 1.39±0.03^cdA^ | 1.31±0.01^eB^ | 1.19±0.03^bC^ | 1.32±0.04^deB^ |
| 2 h | 1.37±0.03^cdeA^ | 1.19±0.04^abB^ | 1.41±0.03^cdA^ | 1.35±0.03^deA^ | 1.16±0.03^bB^ | 1.35±0.03^deA^ |
| 3 h | 1.33±0.05^deA^ | 1.21±0.06^abB^ | 1.41±0.06^cdA^ | 1.34±0.07^deA^ | 1.16±0.03^bB^ | 1.35±0.06^deA^ |
| 4 h | 1.38±0.05^cdeAB^ | 1.24±0.04^abBC^ | 1.47±0.11^bcA^ | 1.39±0.08^cdeA^ | 1.21±0.03^abC^ | 1.41±0.07^cdA^ |
| 5 h | 1.34±0.05^deAB^ | 1.19±0.05^abBC^ | 1.44±0.13^cA^ | 1.37±0.09^deA^ | 1.15±0.03^bC^ | 1.42±0.12^cdA^ |
| 6 h | 1.55±0.09^bcA^ | 1.18±0.05^abB^ | 1.48±0.12^bcA^ | 1.43±0.09^cdeA^ | 1.14±0.03^bB^ | 1.49±0.15^bcdA^ |
| 7 h | 1.6±0.16^bA^ | 1.37±0.07^aB^ | 1.66±0.1^bA^ | 1.61±0.07^bA^ | 1.34±0.03^aB^ | 1.66±0.13^bA^ |
| 8 h | 1.44±0.12^bcdAB^ | 1.24±0.07^abBC^ | 1.57±0.12^bcA^ | 1.52±0.09^bcA^ | 1.18±0.03^bC^ | 1.6±0.16^bcA^ |

*Different lowercase letter within each row indicates significant difference, Different uppercase letter within each column indicates significance difference (p<0.05)*
